# Autonomic Nerve Fibers Aberrantly Reinnervate Denervated Facial Muscles and Alter Muscle Fiber Population

**DOI:** 10.1523/JNEUROSCI.0670-22.2022

**Published:** 2022-11-02

**Authors:** Vlad Tereshenko, Dominik C. Dotzauer, Matthias Luft, Joachim Ortmayr, Udo Maierhofer, Martin Schmoll, Christopher Festin, Genova Carrero Rojas, Johanna Klepetko, Gregor Laengle, Olga Politikou, Dario Farina, Roland Blumer, Konstantin D. Bergmeister, Oskar C. Aszmann

**Affiliations:** ^1^Clinical Laboratory for Bionic Extremity Reconstruction, Department of Plastic, Reconstructive, and Aesthetic Surgery, Medical University of Vienna, 1090 Vienna, Austria; ^2^Centers for Biomedical Research, Medical University of Vienna, 1090 Vienna, Austria; ^3^Department of Orthopedics and Trauma Surgery, Medical University of Vienna, Waehringer Guertel 18-20, 1090 Vienna, Austria; ^4^Medical Physics and Biomedical Engineering; ^5^Anatomy and Cell Biology, Medical University of Vienna, 1090 Vienna, Austria; ^6^Department of Bioengineering, Imperial College London, London SW7 2AZ, United Kingdom; ^7^Department of Plastic, Aesthetic, and Reconstructive Surgery, Karl Landsteiner University of Health Sciences, University Hospital, A-3500 Krems an der Donau, Austria; ^8^Department of Plastic, Reconstructive, and Aesthetic Surgery, Medical University of Vienna, 1090 Vienna, Austria

**Keywords:** autonomic nervous system, facial muscles, facial nerve, muscle fiber types, parasympathetic reinnervation, sensory protection

## Abstract

The surgical redirection of efferent neural input to a denervated muscle via a nerve transfer can reestablish neuromuscular control after nerve injuries. The role of autonomic nerve fibers during the process of muscular reinnervation remains largely unknown. Here, we investigated the neurobiological mechanisms behind the spontaneous functional recovery of denervated facial muscles in male rodents. Recovered facial muscles demonstrated an abundance of cholinergic axonal endings establishing functional neuromuscular junctions. The parasympathetic source of the neuronal input was confirmed to be in the pterygopalatine ganglion. Furthermore, the autonomically reinnervated facial muscles underwent a muscle fiber change to a purely intermediate muscle fiber population myosin heavy chain type IIa. Finally, electrophysiological tests revealed that the postganglionic parasympathetic fibers travel to the facial muscles via the sensory infraorbital nerve. Our findings demonstrated expanded neuromuscular plasticity of denervated striated muscles enabling functional recovery via alien autonomic fibers. These findings may further explain the underlying mechanisms of sensory protection implemented to prevent atrophy of a denervated muscle.

**SIGNIFICANCE STATEMENT** Nerve injuries represent significant morbidity and disability for patients. Rewiring motor nerve fibers to other target muscles has shown to be a successful approach in the restoration of motor function. This demonstrates the remarkable capacity of the CNS to adapt to the needs of the neuromuscular system. Yet, the capability of skeletal muscles being reinnervated by nonmotor axons remains largely unknown. Here, we show that under deprivation of original efferent input, the neuromuscular system can undergo functional and morphologic remodeling via autonomic nerve fibers. This may explain neurobiological mechanisms of the sensory protection phenomenon, which is because of parasympathetic reinnervation.

## Introduction

The locomotor system is essential to move the body, maintain posture, and perform various highly specialized motor tasks (Gatesy and Middleton, 1997; Filimon, 2010). Loss of the motor function can occur because of lesions of upper or lower motor neurons or peripheral neuropathic disorders of different etiologies. These nerve lesions, wherever they manifest, have a substantial negative impact on the quality of a patient's life (Schwartzman et al., 2001; Bergmeister et al., 2020). Untreated motor nerve lesions lead to functional deficits, which often must be surgically treated to restore muscle function.

Despite well-established therapeutic concepts for peripheral nerve injuries (Mackinnon and Novak, 1999; Lee and Wolfe, 2000), many clinical scenarios do not allow a primary nerve repair (Grinsell and Keating, 2014). The slow rate of nerve regeneration, limited resources of donor nerves, and especially the lack of trophic support in long nerve grafts compromise the functional outcome after complex nerve lesions (Campbell, 2008). Because of extensive fibrosis and fatty degeneration of muscle tissue following prolonged muscle denervation, the capacity for functional restoration becomes limited and eventually impossible (Fu and Gordon, 1995; Kobayashi et al., 1997). Sensory nerve transfer for muscle reinnervation is believed to prevent irreversible atrophy of a muscle preceding the motor reinnervation (Bain et al., 2001; Li et al., 2013; Cederna et al., 2015). Nevertheless, the underlying neurobiology of this phenomenon is poorly understood (Zhao et al., 2004).

The motor function of a muscle relies on the integrity of the motor unit with its essential components (Lichtman and Sanes, 2003; Li et al., 2018). The neuromuscular junction (NMJ) is known to be a dynamic entity with remarkable plastic capacities, which is able to remodel as a consequence of specific requirements of ingrowing axons (Nishizawa et al., 2006; Arnold et al., 2014; Ouanounou et al., 2016). In the native state, somatic efferent fibers are indispensable for the functioning of NMJ (Sanes and Lichtman, 1999). Rewiring motor nerve fibers from one motor neural source to another target muscle has been shown to be a successful approach in restoration of motor function (Aszmann et al., 2015; Bergmeister et al., 2019; Ortiz-Catalan et al., 2020). The effect of different neuronal sources (e.g., somatic efferent and visceral efferent axons) on the reinnervation of an alternate target organ is scarcely explored (Langley and Anderson, 1904; Brushart, 1988).

**Figure 1.**
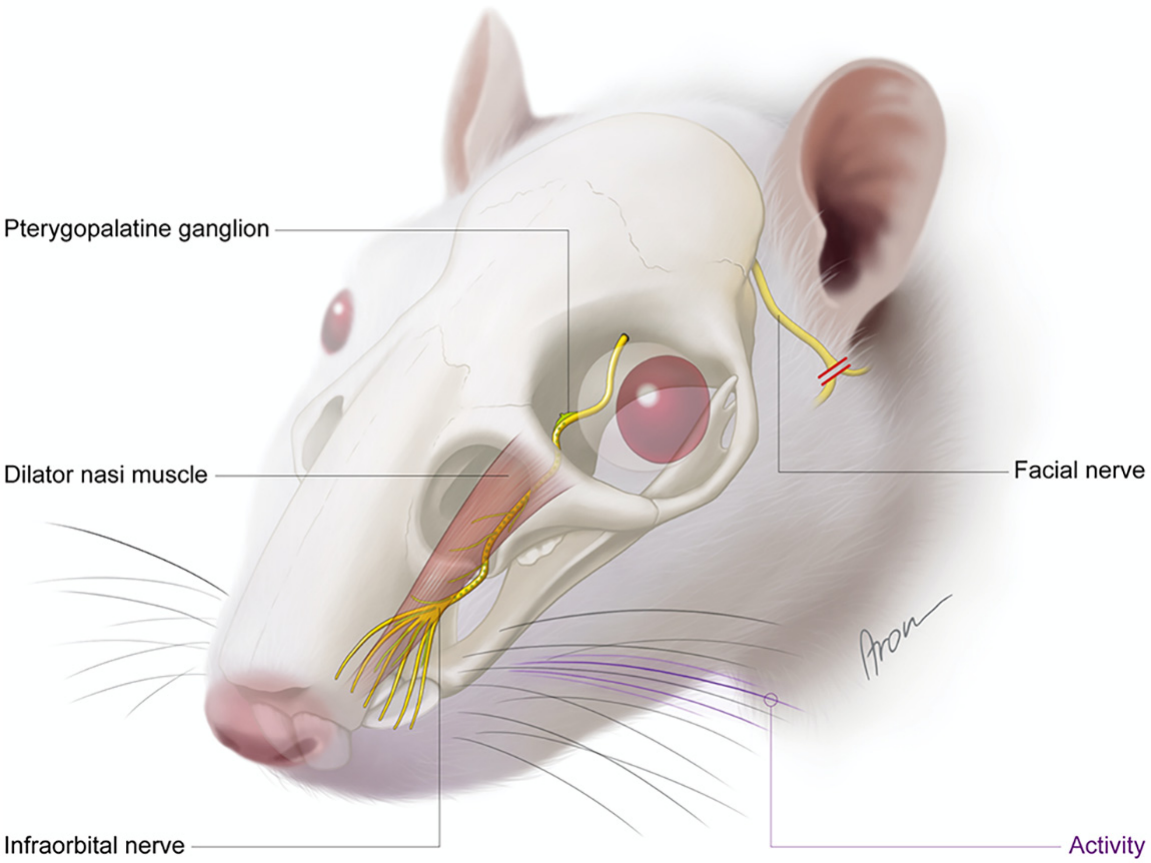
Schematic illustration of aberrant parasympathetic reinnervation of denervated facial muscles. Following facial nerve transection, ipsilateral whisker pad showed spontaneous movement 12 weeks after the denervation. Harvested dilator naris muscle showed muscle fiber change after denervation. The reinnervating fibers were traced to the parasympathetic neural source in the pterygopalatine ganglion. The route of the parasympathetic fibers was established by electrophysiological testing via the sensory infraorbital nerve.

In this study we investigated the neurobiological mechanisms behind the phenomenon of spontaneous functional restoration of facial muscles in the rat. Parasympathetic nerve fibers regenerated via the infraorbital nerve to the facial muscles establishing functional NMJs, which resulted in a change of muscle fiber type and subsequent functional recovery (Fig. 1). This demonstrates that parasympathetic fibers have the capacity of innervating denervated skeletal muscles.

## Materials and Methods

### Animals and study design

Forty Sprague Dawley rats (male, age 8–10 weeks, weighing ∼400 g) were used in this study. The resection procedure of the facial nerve was performed in all rats as described previously (Tetzlaff et al., 1988; Tereshenko et al., 2021). Video assessment of the movement of the whiskers was performed 2, 8, 12, and 16 weeks after the surgery (*n* = 15). An additional group of unoperated rats was included to assess the whisker movement score (*n* = 15). The facial muscles on the denervated and contralateral healthy sides were harvested 12 weeks after the surgery for the whole-mount immunofluorescent staining (*n* = 15). Retrograde labeling was performed by means of intramuscular application of the retrograde tracer (*n* = 5) 12 weeks after the surgery. Qualitative assessment of the neural pathway for reinnervating axons was performed by stimulating the infraorbital nerve 12 weeks after denervation (*n* = 5). Subsequently, muscles from operated and healthy sides were harvested for muscle fiber type staining. Approval was obtained from the ethics committee of the Medical University of Vienna and the Austrian Ministry for Research and Science (reference number, Austrian Federal Ministry of Science, Research, and Economy 66.009/0302-V/II/3b/2019).

### Surgery

All surgeries were performed under standardized conditions. Ketamine (100 mg/kg) and xylazine (5 mg/kg) were injected intraperitoneally preoperatively. Anesthesia was induced by inhalation of isoflurane via an endotracheal tube, and subcutaneous piritramide injections (0.3 mg/kg) were administered for analgesia, which allowed for uneventful surgery. For postoperative analgesia, piritramide and glucose were added to the drinking water (two ampules Dipidolor equaling 30 mg piritramide plus 10 ml 10% glucose solution in 250 ml drinking water) for 5 postoperative days.

The facial nerve main trunk and auricular posterior nerve were transected distal to the stylomastoid foramen as described previously (Tereshenko et al., 2021). Briefly, the facial nerve and auricular posterior nerve were exposed via a preauricular incision and dissected toward their distal branches. The main trunk of the facial nerve was transected 1 mm distal to the stylomastoid foramen followed by transection of a 5 mm nerve segment to the proximal ends of all distal branches. Conclusively, the proximal and distal nerve stumps were cauterized to prevent spontaneous reinnervation.

### Functional assessment of whisker movement

Video recordings of whisker movements were performed in unrestrained rats 2, 8, 12, and 16 weeks following surgery (*n* = 15) as well as in unoperated rats (*n* = 15). Video images of whisking behavior were sampled with a digital camera (12 megapixel, focal length 1.8) at 240 frames/s. For the functional assessment of the active exploration, the operated side was compared with the contralateral side using a 5-point score as follows: 0 = no movement, 1 = barely detectable movement, 2 = less significant movement, 3 = significant but asymmetric movement, 4 = symmetric movement (Farrag et al., 2007; Sun et al., 2011). The recovered muscle tone was analyzed by means of the whisker biometrics (*n* = 9). The static whisker position has been measured in the active explorative state right in the middle between maximal protraction and retraction. The images of whisker position were captured and selected with Adobe Photoshop CS6 (version 13.0). The geometrical model was adapted from the noninvasive video analysis by Tomov et al. (2002) and consisted of three reference points, (1) a point in the median sagittal line (perpendicular to a line connecting both orbits 2 and 3) at the end of the nose, (2) a point corresponding to the medial angle of the left orbit, and (3) a point corresponding to the medial angle of the right orbit (Guntinas-Lichius et al., 2001, 2002, 2007). Large vibrissae from the A-row were evaluated and depicted by two points, a point at the base and a point on the shaft 10 mm away from the base (Carvell and Simons, 1990). Despite the contralateral muscle hypertrophy on the unoperated side, the comparison between two sides allowed for dynamic evaluation of changes in whisker positioning (Rab et al., 2000; Tomov et al., 2002). This model allowed for evaluation of static muscle tone of the vibrissae by measuring the rostrally open angle between the midsagittal plane and the two-point vibrissa shaft (Ego-Stengel et al., 2019). Selected images were processed, and corresponding angles were measured using Adobe Illustrator (version 6.2) by one independent investigator (J.O.).

### Whole-mount muscle analysis

For the facial muscle harvesting, the rats were fixated via left ventricular perfusion under deep anesthesia with 400 ml of NaCl 0.9% followed by 400 ml of 4% diluted PFA in 0.1 m PBS. After perfusion, the facialis muscles including the dilator nasi (MDN) and levator labii superioris (LLS) muscles were dissected from the denervated and control side and stored at 4°C in PBS containing 0.05% sodium azide to avoid bacterial contamination. Before immunolabeling, muscle whole mounts were shock frozen in ice-cold 2-methylbutane at −80°C and immediately thawed. Afterward, tissue was incubated overnight in PBS containing 1% Triton X-100 (PBS-T) at room temperature. After this procedure. immunolabeling started. Muscles underwent whole-mount immunofluorescence staining using the following neuronal markers: chicken anti-neurofilament (1:2000; catalog #AB5539, Millipore; RRID:AB_11212161), goat anti-choline acetyl transferase (anti-ChAT; 1:100; catalog #AB144P, Millipore; RRID:AB_2079751), mouse anti-synaptophysin (1:200; catalog #ab94786, Abcam; RRID:AB_94786). Anti-neurofilament is a pan-neuronal marker. Anti-ChAT visualizes cholinergic axons, anti-synaptophysin synaptic vesicles, and anti-GAP43 nerve fibers during development and regeneration, and α-bungarotoxin is a snake venom that labels acetylcholine receptors. To visualize muscle fibers, we used phalloidin (1:200), a mushroom toxin that binds to actin filaments.

#### Triple-fluorescence staining

Four combinations of triple-labeling were used, (1) anti-neurofilament, α-bungarotoxin, and phalloidin; (2) anti-neurofilament, anti-ChAT, along with α-bungarotoxin; (3) anti-neurofilament, anti-synaptophysin, and α-bungarotoxin; and (4) anti-neurofilament, anti-GAP43, along with α-bungarotoxin.

Before antibody application, the tissue was blocked for 2 h with 10% normal goat serum (staining combination 1, 3, and 4) or 10% normal rabbit serum (staining combination 2). Then the tissue was incubated for 48 h with the primary antibodies diluted in PBS containing 1% PBS-T. Following extensive washing in PBS-T, tissue was incubated for 6 h with the secondary antibodies along with phalloidin or α-bungarotoxin. Finally, the tissue was rinsed again and mounted in 60% v/v glycerin plus 40% PBS. A more detailed description of immunolabeling of muscle whole mounts is provided by Blumer et al. (2020). For negative controls, primary antibodies were omitted, and secondary antibodies were used alone. In all cases, the omission of the primary antibodies resulted in a complete lack of immunostaining.

#### Confocal laser scanning microscope analysis

Fluorescently labeled muscle whole-mounts were analyzed with a confocal laser scanning microscope (CLSM; Olympus FV3000). A series of virtual CLSM sections of 1 µm thickness were cut through the structures of interest. Each section was photodocumented with a 1024 × 1024 pixel resolution, and 3D projections were rendered using ImageJ software (National Institutes of Health). Triple-colored images were generated using lasers with excitation wavelengths of 488, 568, and 633 nm.

#### Quantification analysis

The number of NMJs was quantified in the three-dimensional images acquired by confocal imaging. The images were acquired in the middle of the MDN muscle to quantify as many NMJs as possible. The images were evaluated using software tools from Adobe Photoshop CS6 (version 13.0) by one independent investigator (V.T.). The surface area of the image was measured, and the total number of NMJs, innervated NMJs as well as polyinnervated NMJs, have been quantified in the control and operated MDNs (*n* = 8). The number of NMJs was extrapolated on a 1 mm^2^ of the muscle tissue.

### Muscle fiber type composition

Muscle fiber types were assessed on both operated and contralateral MDN. After electrophysiological assessment, the entire MDN was carefully dissected and removed. Afterward, muscles were embedded in optimal cutting temperature compound (Tissue-Tek) using liquid-nitrogen-cooled isopentane and stored at −80°C for 24 h. Cross sections 10 µm thick of the embedded muscles were then obtained. A modified version of a previously described immunofluorescent protocol was used (Bergmeister et al., 2016, 2019). First, Wheat germ agglutinin conjugated Alexa Fluor 594 (1:250) was applied for 10 min to stain the muscle fiber membrane. Primary antibodies diluted in 0.1 m PBS with 10% goat serum against myosis heavy chain (MHC)-I (BA-F8; 1:50), MHC-IIa (SC-71; 1:600), and MHC-IIb (BF-F3; 1:100) were used and applied for 60 min (Developmental Studies Hybridoma Bank). Afterward, the secondary antibodies against Alexa Fluor 633 immunoglobulin G2b (IgG2b; 1:250), Alexa Fluor 488 IgG1 (1:250), and Alexa Fluor 555 IgM (1:250) were applied for 60 min (Life Technologies). Entire cross sections of the muscle were acquired using a whole-slide scanner (Vectra Polaris, Akoya Biosciences) and subsequently analyzed using the Halo imaging analysis platform version 3.2.1851.421 (Indica Labs) by one independent investigator (M.L.).

### Retrograde labeling

The neuronal sources of the parasympathetic fibers responsible for the reinnervation were traced using an intramuscular retrograde labeling procedure (*n* = 5). The facial muscles (MDN and LLS) were exposed 12 weeks following the denervation procedure. Using a 10 µl Hamilton microsyringe (catalog #7635-01, Hamilton Bonaduz) with a small hub removable needle (30 ga; catalog #7803-07, Hamilton Bonaduz), 3 µl of 2% Fast Blue (catalog #17740-5, Polysciences) was injected in both muscles. To prevent any leakage from the muscles, the needle was left within the muscle for 30 s followed by slow withdrawal. One week after the tracer application, left ventricular perfusion was performed as described above. The brain and the pterygopalatine ganglion on both sides were harvested and stored in 0.1 m PBS for 48 h. After dehydration in increasing sucrose/PBS solutions (10%, 25%, 40%), the samples were cryofixed and cut using a cryostat (Leica), the brainstem in 50 µm and the ganglia in 15 µm. The number of labeled neurons was quantified by one trained observer (V.T.). To avoid double counting of perikarya, the average nucleolus diameter was calculated to apply the Abercrombie correction.

### Electrophysiology

The neural pathway of the parasympathetic fibers was determined by electrical stimulation of the infraorbital nerve and evaluation of the ipsilateral whisker movement (Movie 1). Electrical stimulation was delivered by a custom-built pulse generator (MiniVStim 18B, Competence Team for Implanted Devices, Center of Medical Physics and Biomedical Engineering, Medical University of Vienna) to elicit 10 short contractions (500 ms ON, 500 ms OFF). The stimulator generated symmetric, biphasic, rectangular, charge-balanced current pulses (amplitude, 8 mA; phase width, 2 ms) at a stimulation frequency of 70 Hz. The anode, a subdermal stainless steel needle electrode (diameter, 0.4 mm; Rhythmlink International) was placed into the middle portion of the infraorbital nerve. A surgical stainless steel hook was used as the anode. The movement of the whiskers was recorded via a standard camera.

Movie 1. Experimental setting for the electrophysiological assessment. Intraoperative stimulation of the exposed infraorbital nerve (blue vessel loop) was performed under anesthesia. The isolated stimulation of the infraorbital nerve resulted in whisker movement. 10.1523/JNEUROSCI.0670-22.2022.video.1

### Statistical analysis

In the group that underwent retrograde labeling, descriptive statistics were used for further analysis. The acquired data are presented either as absolute and relative values or as means and SDs. Whisker movement score (categorical variable) among different groups (2, 8, 12, and 16 weeks) was assessed using a Kruskal–Wallis test; the operated groups were compared with the control group using a one-sample Wilcoxon test. Results with a *p* value of <0.05 were considered significant. For the assessment of the angle of vibrissal orientation, normal distribution was checked with the Kolmogorov–Smirnov test and showed a Gaussian distribution in all groups. Angle of vibrissal orientation was compared between control and operated sides over the time course using a two-way ANOVA test. Angle of vibrissal orientation was compared with control and operated sides within each time period using a paired *t* test. The difference of vibrissal angle between control and operated sides (2, 8, 12, and 16 weeks) was compared over time using a one-way ANOVA test. Results with a *p* value of <0.05 were considered significant. In the NMJ assessment group, normal distribution was checked with the Shapiro–Wilk test. This showed a Gaussian distribution in all groups. The number of NMJs in control and operated muscles were compared using a paired *t* test. Results with a *p* value of <0.05 were considered significant. The number of muscle fibers as well as the number of cell nuclei between control and operated muscles were compared using a paired *t* test.

### Data availability

All data are available in the main text of this article or in the supplementary video (Movie 1).

## Results

### Spontaneous whisker movement after facial nerve denervation

**Figure 2.**
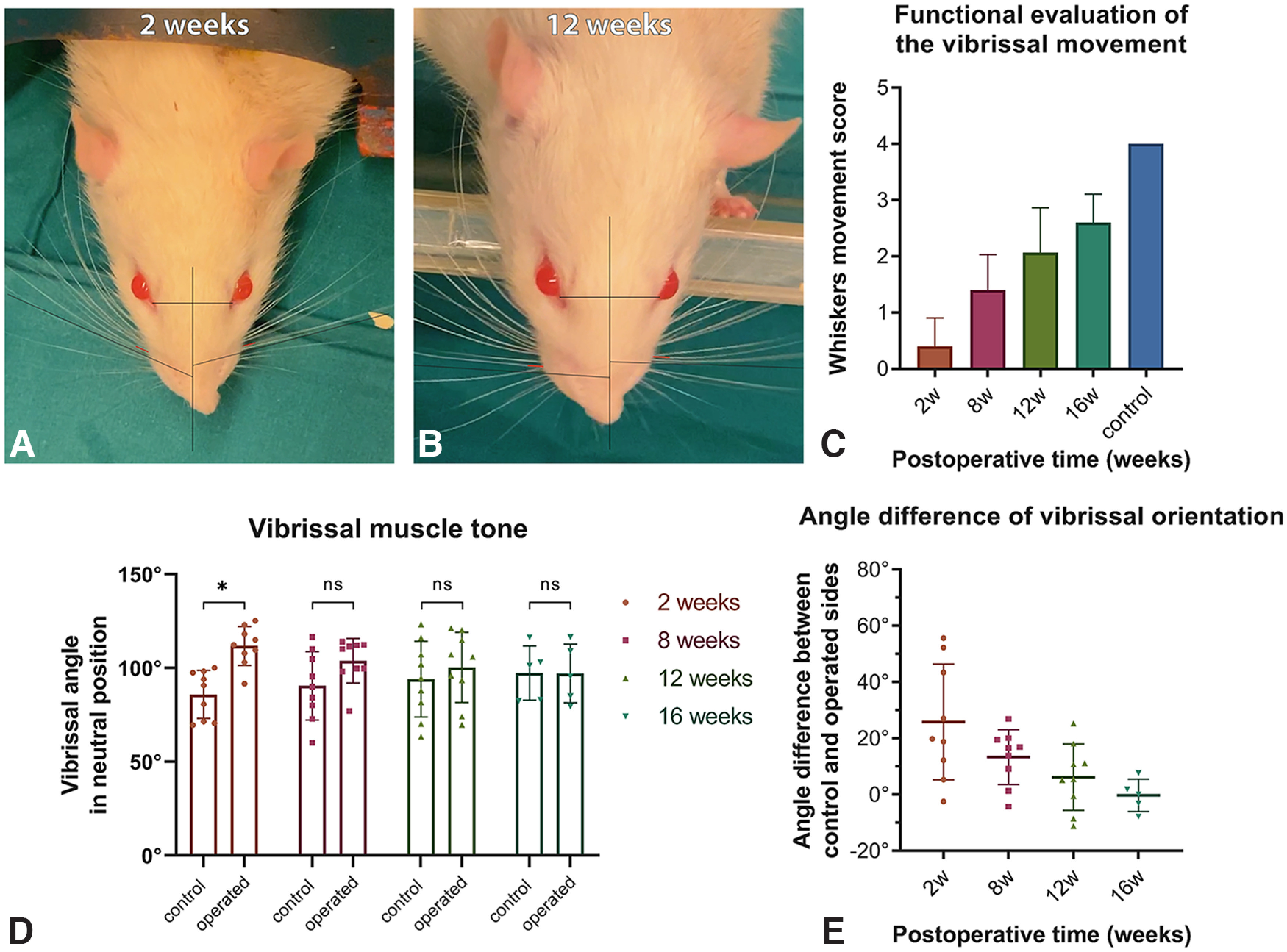
Functional assessment of the recovered whisker movement. ***A***, Whisker orientation assessment 2 weeks after surgery. ***B***, Whisker orientation assessment 12 weeks after surgery. Whisker movement score 2 weeks [0 interquartile range (IQR; 0–1)], 8 weeks [1 (IQR 1–2)], 12 weeks [2 (IQR 1–3)] and 16 weeks [3 (IQR 2–3)] after surgery (*n* = 15) as well as the control group [4 (IQR 4–4); *n* = 15]. Whisker movement score was significantly different among all operated groups [2, 8, 12, and 16 weeks; *p* < 0.001, H(3) = 38.2]. ***C***, None of the rats achieved the highest whisker movement score (score = 4) at 2, 8, 12, or 16 weeks after surgery compared with the control group [*p* < 0.01, W(1) = 120.0 for all groups]. Comparison of whisker orientation angle 2, 8, 12, and 16 weeks after surgery between control and operated sides was performed using a two-way ANOVA test. No significant difference was observed over the time course (*p* = 0.15, *F*_(3,56)_ = 1.87). In the 2 and 8 weeks group there was a statistically significant difference between operated and control sides, 111.8 ± 10.4° versus 86 ± 12.8° (*p* < 0.01, *t*_(8)_ = 3.8) and 103.9 ± 11.9° vs 90.5 ± 18.3° (*p* < 0.01, *t*_(8)_ = 4.1). ***D***, No difference was found between operated and control sides after 12 and 16 weeks, 100.4 ± 18.7° versus 94.1 ± 20.2° (*p* = 0.15, *t*_(8)_ = 1.58) and 97.2 ± 15.6° versus 97.35 ± 14.5° (*p* = 0.94, *t*_(4)_ = 0.08), respectively. Angle differences between control and operated sides were compared between 2, 8, 12, and 16 weeks groups. ***E***, One-way ANOVA revealed significant reduction of the angle difference between control and operated sides from 2 until 16 weeks after surgery (*p* = 0.008, *F*_(3,28)_ = 4.79).

Motor function of the whiskers was spontaneously restored following facial nerve resection. The mystacial whisker pad is normally innervated by the buccal and marginal mandibular branches of the facial nerve (Henstrom et al., 2012). Functional video assessment showed partly restored whisker movement 12 weeks after the facial nerve neurotomy. Most of the rats showed an asymmetric but significant whisker movement (score, 3) 16 weeks after surgery. The whisker movement score was significantly different among all operated groups (2, 8, 12, and 16 weeks [*p* < 0.001, H-statistic H(3) = 38.2]. None of the rats achieved the highest whisker movement score (score, 4) at 2, 8, 12, or 16 weeks after surgery compared with the control group [*p* < 0.01, W-statistic W(1) = 120.0 for all groups; Fig. 2].

The muscle tone was assessed by measuring the angle of vibrissal orientation in the neutral position. The angle of the vibrissae on the operated side (111.8 ± 10.4°) was significantly higher (i.e., lower muscle tone) compared with that of the control side (86 ± 12.8°) 2 weeks (*p* < 0.01, *t*_(8)_ = 3.8); 8 weeks (103.9 ± 11.85° vs 90.5 ± 18.3°, *p* < 0.01, *t*_(8)_ = 4.1) after surgery (Fig. 2*A*,*B*,*D*). From the 12th to the 16th week after denervation there was no significant difference between the operated and control sides (*p* = 0.15, *t*_(8)_ = 1.58 for 12 weeks; *p* = 0.94, *t*_(4)_ = 0.08 for 16 weeks; Fig. 2*C*). A two-way ANOVA test revealed no statistically significant angle difference between two operated and control sides over the time course (*p* = 0.15, *F*_(3,56)_ = 1.87). The angle difference between control and operated sides reduced significantly from 2 until 16 weeks after surgery (*p* = 0.008, *F*_(3,28)_ = 4.79; Fig. 2*D*).

### Parasympathetic fibers form functional NMJs

**Figure 3.**
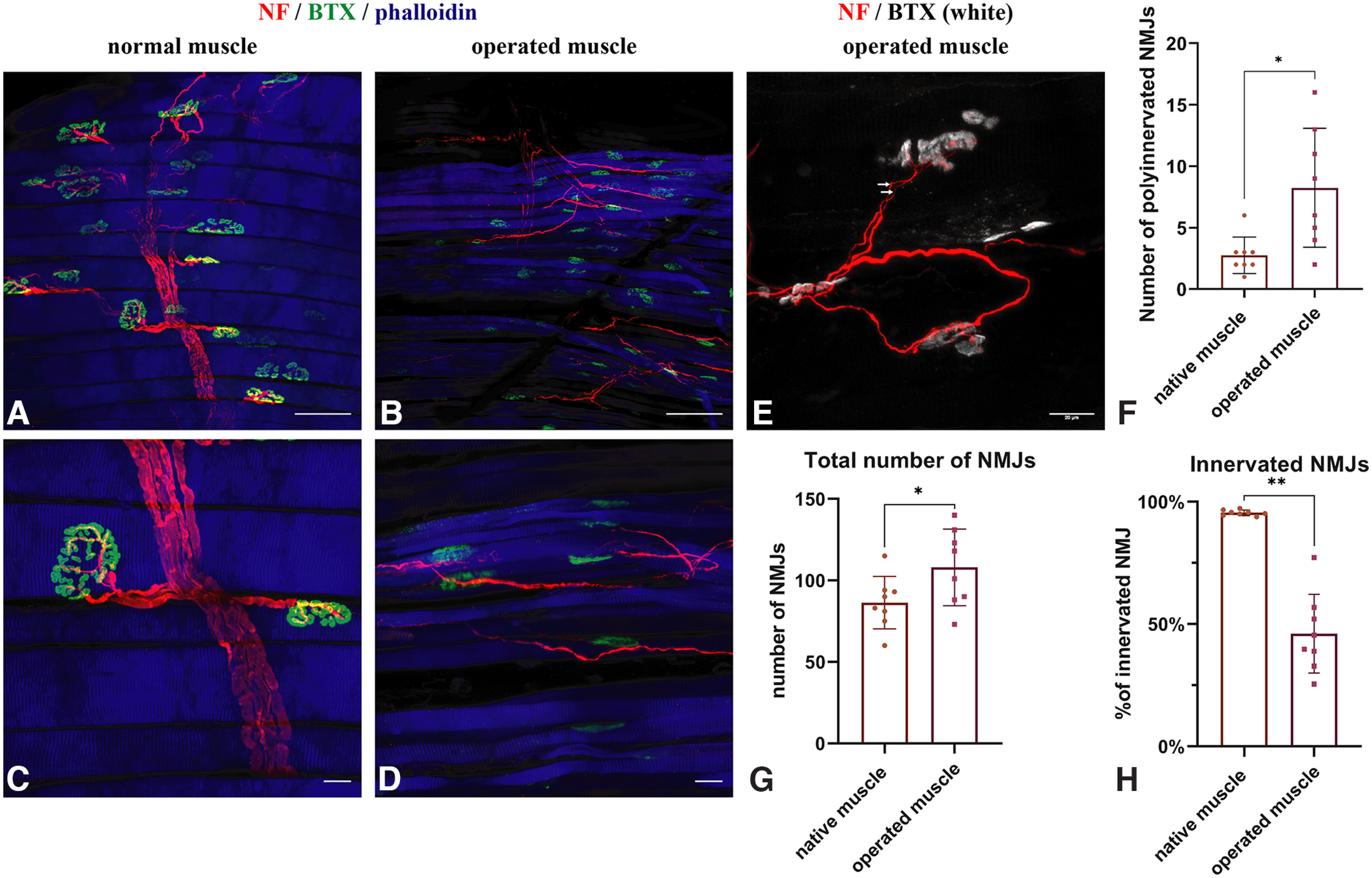
Whole-mount staining of the NMJs and the reinnervation pattern. ***A***, Whole-mount staining of the control MDN using anti-NF antibody, α-bungarotoxin (BTX), and phalloidin. Scale bar, 100 µm. ***B***, In contrast, thin fibers reach the dysmorphic NMJs in a denervated MDN. Scale bar, 100 µm. ***C***, Clear innervation of the NMJs in the control muscle is demonstrated via thick fibers. Scale 20 µm. ***D***, Reinnervation of the dysmorphic and small NMJs is demonstrated in the operated muscle. Scale bar, 20 µm. ***E***, An example of a NMJ reinnervated via two thin axons (2 white arrows) in an operated muscle. Scale bar, 20 µm. ***F***, Number of polyinnervated NMJs is not significantly higher between control and operated muscles (paired *t* test, *p = 0.01). ***G***, The total number of NMJs was significantly higher in the operated group (paired *t* test, *p = 0.0154). ***H***, Number of innervated NMJs was significantly higher in the control muscle (paired *t* test, **p = 0.01).

Whole-mount staining of control muscles revealed axons with a large diameter that established synaptic contacts with α-bungarotoxin-labeled acetylcholine receptors (Fig. 3*A*,*C*). In contrast, whole-mount staining of the denervated facial muscles (Fig. 3*B*,*D*,*E*) revealed an abundance of thin nerve fibers suggesting newly ingrown axons. The LLS as well as the MDNs demonstrated anti-neurofilament (NF)-positive axons sprouting toward α-bungarotoxin-labeled acetylcholine receptors (Fig. 3*B*,*D*,*E*). Some axons established contacts with the postsynaptic acetylcholine receptors (Fig. 3*D*,*E*). Compared with the normal muscle, NMJs in the operated muscles were smaller and showed a dysmorphic structure. The total number of NMJs was significantly higher in the operated muscles compared with the control side (108.0 ± 23.5 vs 86.4 ± 16.0; *p* = 0.015, *t*_(7)_ = 3.2). The number of polyinnervated NMJs (two or more axons) was higher in the operated group as well (8.3 ± 4.8 vs 2.8 ± 1.5; *p* = 0.01, *t*_(7)_ = 3.5; Fig. 3*E–G*; Guntinas-Lichius et al., 2005). The portion of innervated NMJs was significantly higher in the control muscle (95.5 ± 1.1 vs 46.1 ± 16.1; *p* = 0.01, *t*_(7)_ = 3.5; Fig. 3*H*).

**Figure 4.**
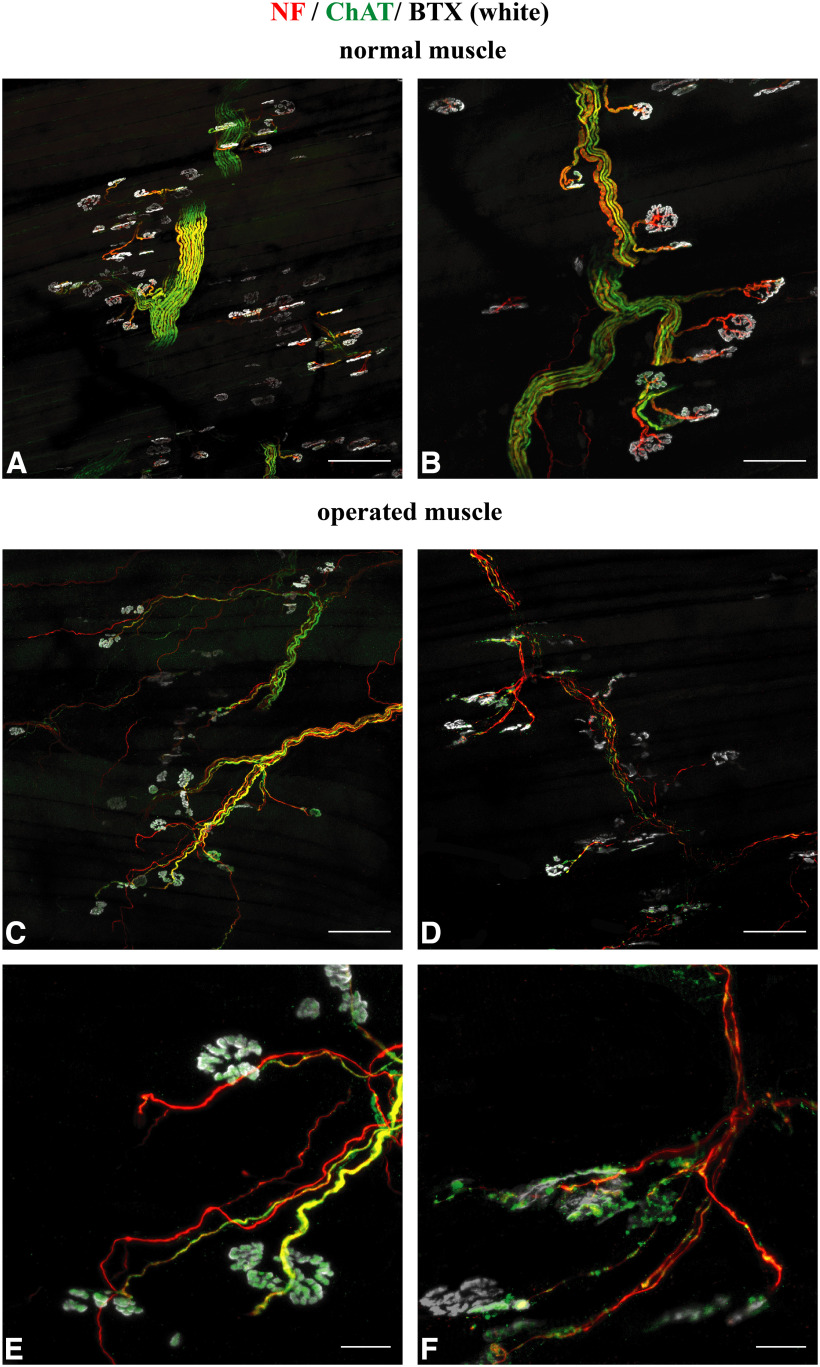
Reinnervation pattern via parasympathetic fibers of denervated facial muscles. ***A***, ***B***, Whole-mount staining showed the specific cholinergic nature of somatic efferent fibers in the control MDN. Scale bars: ***A***, 200 µm; ***B***, 100 µm. ***C***, ***D***, Magnified image shows somatic efferent fibers with thick diameters Reinnervation pattern of the denervated MDN demonstrates thin cholinergic fibers reaching to the NMJs. Scale bar, 100 µm. ***E***, ***F***, Magnified images of the ChAT-positive parasympathetic fibers reaching to the NMJs. Scale bar, 20 µm.

**Figure 5.**
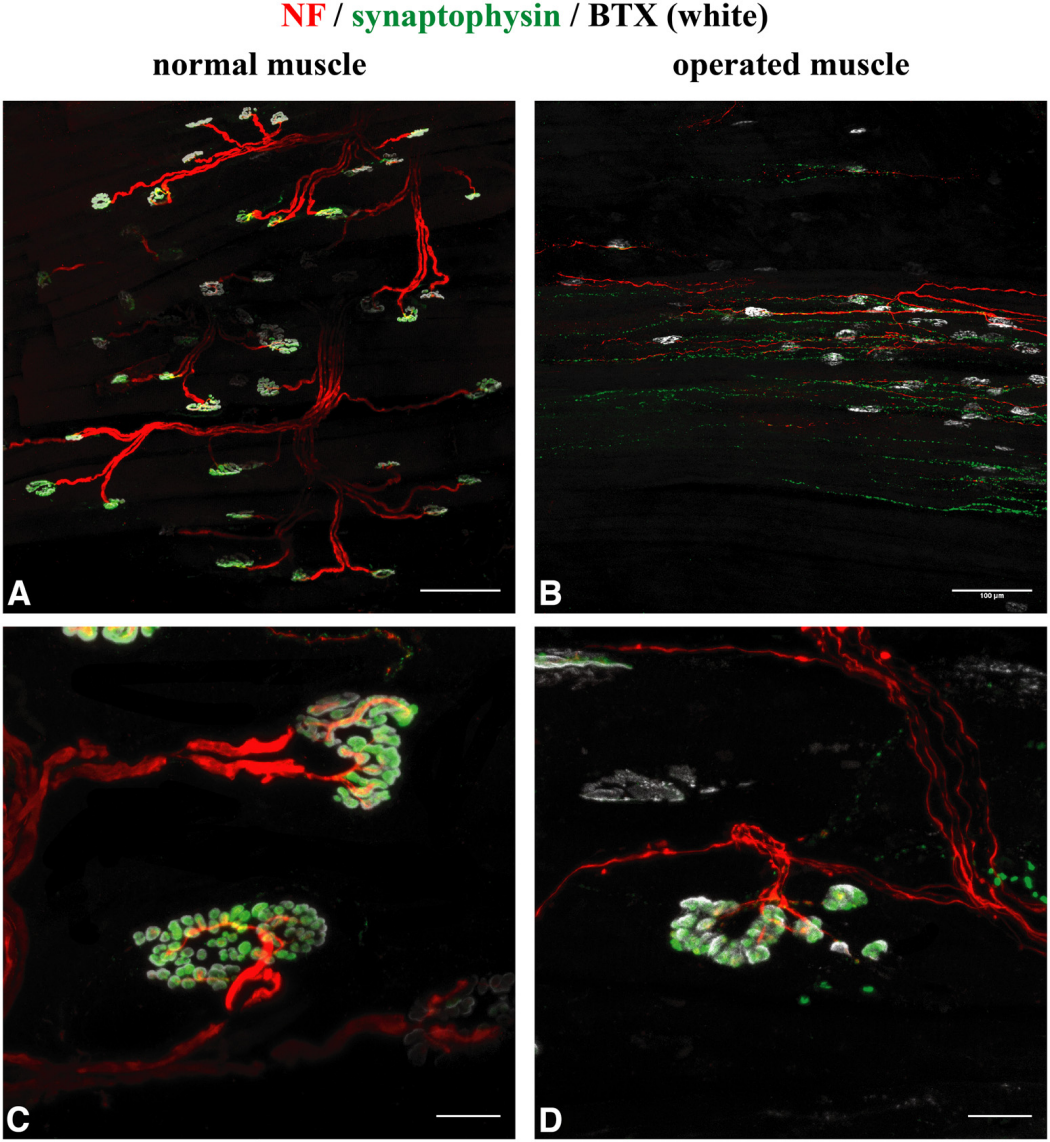
Whole-mount staining reveals functional connectivity of the NMJs and newly sprouting nature of the axons. ***A***, ***C***, NMJs of the control MDN. All NMJs exhibit synaptophysin immunoreactivity. Scale bars: ***A***, 100 µm; ***C***, 20 µm. ***B***, ***D***, NMJs in a denervated MDN. Only NMJs with adjacent axons are synaptophysin positive indicating the ability of the nerve endings to produce functional connections with the muscle fiber. Scale bars: ***B***, 100 µm; ***D***, 20 µm.

Using anti-ChAT as a specific marker for cholinergic axons, whole-mount staining of control muscles revealed that axons contacting the muscle fibers had a cholinergic phenotype (Fig. 4*A*,*B*). In denervated muscles, axons contacting the muscle fiber were cholinergic as well (Fig. 4*C–F*). The thin diameter of the nerve fibers indicated their parasympathetic nature. To evaluate that newly formed nerve fibers establish functional connections with the muscle fibers, staining with anti-synaptophysin was done. Synaptophysin is a synaptic vesicle protein and present at the presynaptic site of neurons. It is supposed that synaptophysin plays a role in neurotransmitter release (De Camilli et al., 1988). In control muscles, all α-bungarotoxin NMJs exhibited synaptophysin signals (Fig. 5*A*,*C*). In operated muscles, synaptophysin was associated with reinnervated NMJs, whereas NMJs without axonal support exhibited no synaptophysin signal (Fig. 5*B*,*D*). This suggests that reinnervated NMJs are highly specialized and capable of synaptic transmission (Valtorta et al., 2004).

### Muscle fiber composition changes following autonomic reinnervation

**Figure 6.**
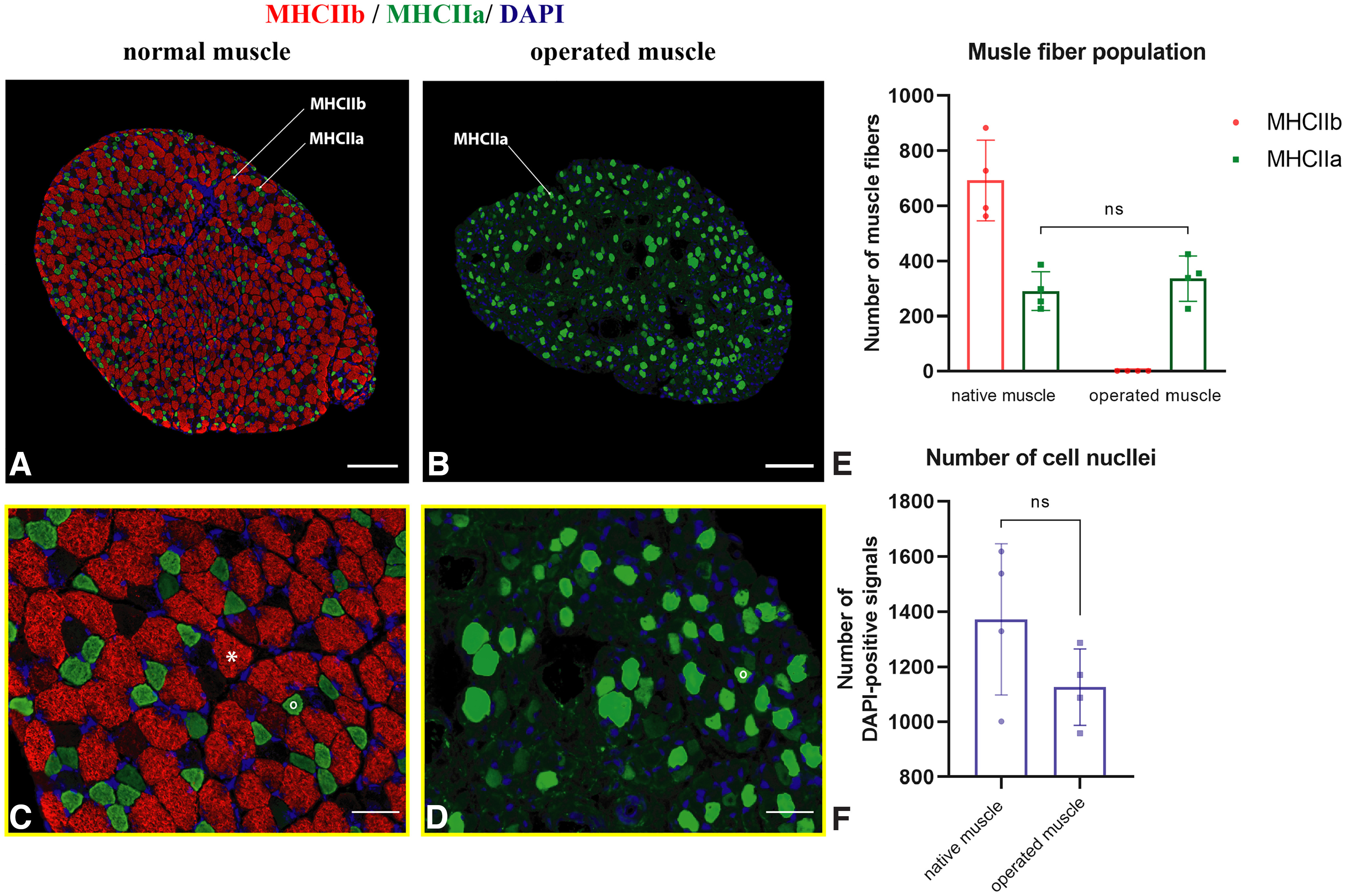
Muscle fiber population of the dilator nasi muscle. ***A***, ***C***, Control MDN displays a mixed muscle fiber population of 692 ± 147 MHCIIb (*red) and 291 ± 71 MHCIIa (°green) types. ***B***, ***D***, Autonomically reinnervated MDN consist exclusively of the MHCIIa type (336 ± 83). No centrally located DAPI-positive signals were observed in the operated muscle. Scale bars: ***A***, ***B***, 400 µm; ***C***, ***D***, 50 µm. ***E***, No statistically significant difference was observed regarding the number of MHCIIa type fibers between operated and control muscles (paired *t* test, *p* = 0.57). ***F***, The nuclei number was not statistically different between control and operated muscles (paired *t* test, *p* = 0.28).

The muscle fiber composition changed after the spontaneous autonomic reinnervation. The original muscle fiber population of the MDN consists of the MHCIIa (291 ± 71) and MHCIIb (336 ± 83) types. Compared with the extremity skeletal muscles, no MHCI (slow) fibers were found in the MDN (Bergmeister et al., 2019). The muscle fiber population in the reinnervated MDN changed toward purely MHCIIa fiber types, indicating its intermediate twitch nature (Fig. 6). The number of the MHCIIa type in the autonomically reinnervated MDN (336 ± 82) did not significantly differ from that of the control side (290 ± 70; *p* = 0.57, *t*_(3)_ = 0.62; Fig. 6*E*). The overall number of muscle fibers was lower in the denervated group (336 ± 82) compared with that of the control (983 ± 195; *p* = 0.013, *t*_(3)_ = 5.3).

The number of the cell nuclei in the reinnervated MDN was similar to the control side (1126 ± 137 vs 1371 ± 275; *p* = 0.28, *t*_(3)_ = 1.3; Fig. 6*F*). Moreover, no DAPI-positive signals were found located centrally within the muscle fibers. These findings suggest that the parasympathetic input is sufficient to prevent a chronic denervation process in the denervated MDN (Chargé and Rudnicki, 2004).

### Neuronal sources of the reinnervating fibers

**Figure 7.**
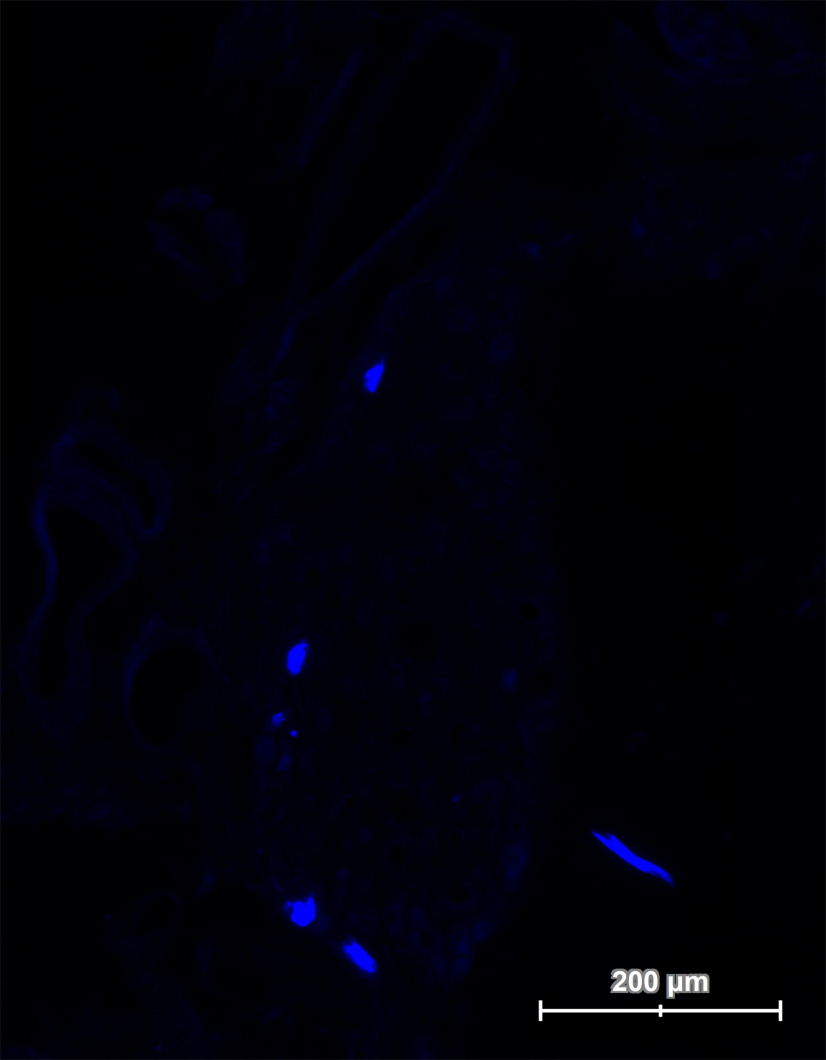
Immunofluorescence image of the retrogradely labeled pterygopalatine ganglion. One week after intramuscular application (dilator nasi and levator labii superioris muscles) of a retrograde tracer (Fast Blue), the ganglion was harvested (*n* = 5). The quantification analysis revealed 46 ± 10 labeled cell bodies within the pterygopalatine ganglion.

The reinnervating parasympathetic axons were back to their origin in the pterygopalatine ganglion (Fig. 7). Following the intramuscular application of the retrograde tracer to the LLS and MDN, no fluorescent signals were found within the brainstem (*n* = 5). Lack of evidence for stained motor neurons within the brainstem suggests that no axons of somatic efferent nature reached the denervated facial muscles. This excludes somatic efferent reinnervation of the denervated facial muscles via any of the motor cranial nerves. The ipsilateral geniculate ganglion did not show any positive signal as well. The pterygopalatine ganglion showed 46 ± 10 labeled cell bodies, confirming their parasympathetic nature (*n* = 5).

### Autonomic nerve fibers reinnervate facial muscles via the infraorbital nerve

The neural route of the parasympathetic fibers was assessed via electrophysiological stimulation (Movie 1). Twelve weeks following the denervation surgery, the whisker movement could be elicited through ipsilateral stimulation of the infraorbital nerve (*n* = 5). No movement of the contralateral whisker pad was observed. This qualitative assessment suggests that parasympathetic axons travel via the infraorbital nerve to the denervated muscles and build new functional neuromuscular junctions.

## Discussion

Different nerve fiber types are believed to be predestined for specific innervation of their target end organs (Lumsden and Davies, 1983; Sanes and Lichtman, 2001). Pathologic conditions, however, may disrupt natural neural connections resulting in dysfunctional reinnervation (Gordon, 2020). The remodeling capacity of some target end organs (e.g., muscles, mechanoreceptors) allows for functional connections with axons from an alternate neuronal source but of the same neuronal quality (Bergmeister et al., 2019; Luft et al., 2021). Yet, the capability of a target organ being reinnervated by a foreign axon type remains largely unknown (Brunelli et al., 2005). The most striking finding of this study is that under deprivation of original somatic efferent input, the facial neuromuscular system undergoes functional and morphologic remodeling via autonomic nerve fibers. We demonstrated the parasympathetic nature behind the spontaneous recovery of whisker movement in a chronic denervation model in the rat. The newly ingrown postganglionic parasympathetic fibers traveled via the infraorbital nerve from the pterygopalatine ganglion to the denervated facial muscles and changed their muscle fiber population to a slower twitching (MHCIIa) type.

Although the neural plasticity of the CNS has been extensively investigated under various pathologic conditions (Cauraugh and Summers, 2005; Kleim and Jones, 2008; Sturma et al., 2020), the plastic capacity of the peripheral nervous system, particularly in the neuromuscular system, is poorly understood (Grinnell and Herrera, 1981). In contrast to post-traumatic cortical plasticity, muscle denervation triggers plastic remodeling, which allows functional reinnervation via other neuronal sources. This phenomenon was originally demonstrated by Flourens in 1828, where upper and lower trunks of the brachial plexus in the rooster were cross-coaptated, resulting in functional cross-reinnervation of the wing muscles (Flourens, 1828). Subsequently, nerve transfer surgeries have become a feasible procedure in experimental and clinical settings because of plastic properties of the skeletal muscles (Oberlin et al., 1994; Bergmeister et al., 2019; Luft et al., 2021). Our findings, however, further expand the understanding of muscular plastic capacity, showing possible functional reinnervation via autonomic sources as well.

The parasympathetic nervous system is a part of the autonomic nervous system regulating visceral organs and blood vessels (Browning and Travagli, 2014). Despite involuntary properties of the parasympathetic system, it resembles the somatic efferent system in many ways. The parasympathetic as well as somatic efferent systems are cholinergic in nature (Sanes and Lichtman, 2001), and the effector of both systems is muscle tissue. However, smooth muscles (targeted by the parasympathetic system) and skeletal muscles (targeted by the somatic efferent system) differ in their ultrastructural morphology, physiology, and embryogenesis (Waite and Li, 1993; Somlyo and Somlyo, 1994; Eddinger, 1998). A previous study reported on the sympathetic axons in the extracranial facial nerve, which highlighted their possible contribution to the maintenance and modulation of neuromuscular junctions (Takeuchi et al., 1993; Tereshenko et al., 2022). One of the intriguing questions is whether the neural input is essential for differentiated properties of the muscle tissue and if it can determine not only the different transcriptome activations but remodel morphologic and physiologic properties of muscle tissue as well (de Medinaceli and Rawlings, 1987; Weng et al., 2018). Effects of redirecting autonomic neural inputs to a skeletal muscle are scarcely explored (Coget and Rousseau, 1983; Flumerfelt et al., 1986; Delorme et al., 1997; Heaton et al., 2014; Gordon, 2020). Interestingly, some studies investigated preganglionic retrograde reinnervation of the skeletal muscle by redirecting the distal stump of the vagus nerve to the sternomastoid muscle without involvement of central neural input (Coget and Rousseau, 1983; Delorme et al., 1997; Gordon, 2020). The lack of neuronal support from the CNS does not allow for voluntary muscular contraction, which questions the purpose of surgically redirecting preganglionic autonomic fibers to a skeletal muscle (e.g., via the vagus or trigeminal nerves) in the clinical routine. However, one study reported that the reinnervated striated muscles changed expression profiles in the redirected motor neuronal sources, indicating retrograde influence of the end organ on the neuronal sources (McWilliam et al., 1995). Others studied autonomic reinnervation of a skeletal muscle from central neuronal sources (Flumerfelt et al., 1986); however, neither functional assessment nor the differentiation between sympathetic and parasympathetic input have been performed. Our data showed specific immunofluorescent evidence of the parasympathetic nature of the sprouting reinnervating fibers (Figs. 4, 5). Moreover, the retrograde tracing trial demonstrated an unambiguous central origin of the postganglionary parasympathetic fibers in the pterygopalatine ganglion. Nevertheless, the pterygopalatine ganglion might not be the single source of the parasympathetic axons. The otic ganglion can serve as another source of the regenerating axons as well. Hence, we showed that postganglionic fibers of the parasympathetic system are capable of establishing functional neuromuscular junctions within a denervated striated skeletal muscle. These findings further increase our understanding of the neuroplastic potential of the autonomic nervous system.

The ability of the parasympathetic system to reinnervate a striated skeletal muscle points out the extended plastic capacity of a skeletal muscle as well. Different muscle fiber types are defined by the MHC isoforms, which are the main determinants for muscular properties (e.g., force–velocity, muscle tone, fatigue resistance, contraction speed, etc.; Schiaffino and Reggiani, 2011). A denervated muscle recapitulates embryonic myogenesis resulting in expression of developmental MHC isoforms (Sartore et al., 1952; Schiaffino and Reggiani, 2011), which are replaced by adult MHC isoforms depending on the reinnervating nerve activity. In case of the muscle reinnervation, muscle fiber differentiation is dictated by a nerve activity-dependent pathway. This results in slow- or fast-twitch-like muscle fiber change corresponding with the firing pattern of reinnervating slow or fast motoneurons (Pette and Vrbová, 1999; Bergmeister et al., 2019). A long-term denervated muscle undergoes a fast-like muscle fiber change triggered by default activation of the fast gene program (Whalen et al., 1990; Esser et al., 1993). Interestingly, the dilator nasi muscle in the rat contains only fast (MHCIIb) and intermediate (MHCIIa) fibers and lacks slow muscle fibers (MHCI; Jergovi et al., 2001), which we confirmed in our study using a new quintuple immunofluorescent staining (Fig. 6). We demonstrated a total muscle fiber change toward MHCIIa with elimination of MHCIIb fibers after the parasympathetic reinnervation, making the dilator nasi a slower muscle. Moreover, functional assessment of the movement of the whiskers showed not only dynamic functional recovery but recovered muscle tone of the facial muscles as well. This suggests that the parasympathetic nervous system remodeled facial muscles into a slower kind, and, hence, is responsible for recovery of the muscle tone. This corresponds with an essential role of the parasympathetic system in muscle tone control of the smooth muscles (Canning, 2006). These findings showed the remodeling adaptability of the denervated facial muscles under reinnervation via alien neural sources (parasympathetic system).

The outlined plastic properties of the neuromuscular system may be considered in the treatment strategies of diverse neuropathological conditions. Long-standing muscle atrophy may become irreversible if no motor reinnervation occurs (Fu and Gordon, 1995). A recent study showed the quantal release of acetylcholine itself activates acetylcholine receptors, prevents muscular atrophy, and favors subsequent muscular reinnervation (Cisterna et al., 2020). This indicates the ability of the parasympathetic fibers to prevent muscular atrophy by remodeling muscle morphology in our study. This phenomenon may be considered as a part of the concept of neuromuscular reconstruction, which is based on the replace like with like principle. Nevertheless, if there is no clinically justifiable option to replace a motor nerve with another one, a so-called sensory protection could become an alternative (Bain et al., 2001, 2008). Using a sensory nerve as a preliminary muscular innervation for subsequent motor reinnervation is a surgically feasible concept, but its fundamental neurobiological processes are not understood. Therefore, this concept is scarcely applied in the clinical routine, but some experimental studies showed sensory protection results in successful muscle atrophy prevention or facilitates motor muscle reinnervation (Hynes et al., 1997; Wang et al., 2001; Li et al., 2013; Placheta et al., 2015; Hosseinian et al., 2018). Because all cutaneous nerves contain autonomic fibers (Roosterman et al., 2006), our findings suggest a possible neurobiological explanation of the sensory protection phenomenon, which is because of the parasympathetic reinnervation. This new paradigm into the sensory protection mechanisms can revolutionize treatment approaches of facial palsy, nerve lesions in the lower extremity, as well as proximal nerve injuries of the brachial plexus. Furthermore, our findings may explain the phenomenon of the spontaneous functional recovery after surgical resection of the facial nerve, which has been anecdotally reported (Martin and Helsper, 1957; Trojaborg and Siemssen, 1972; Hakami and Mosavy, 1976), but the underlying neurobiological mechanisms have remained elusive so far.
